# Fixed and adaptive beamforming improves speech perception in noise in cochlear implant recipients equipped with the MED-EL SONNET audio processor

**DOI:** 10.1371/journal.pone.0190718

**Published:** 2018-01-05

**Authors:** Clemens Honeder, Rudolfs Liepins, Christoph Arnoldner, Hana Šinkovec, Alexandra Kaider, Erich Vyskocil, Dominik Riss

**Affiliations:** 1 Department of Otorhinolaryngology, Head and Neck Surgery, Medical University of Vienna, Vienna, Austria; 2 Center for Medical Statistics, Informatics and Intelligent Systems, Section for Clinical Biometrics, Medical University of Vienna, Vienna, Austria; Medical University Hannover; Cluster of Excellence Hearing4all, GERMANY

## Abstract

**Objective:**

To determine the impact of the fixed and adaptive beamforming technology of the new MED-EL SONNET cochlear implant audio processor on speech perception in noise.

**Methods:**

The study cohort comprises 18 postlingually deafened adult cochlear implant recipients with at least six months of experience. Speech reception thresholds were measured with the Oldenburg Sentence Test in continuous, speech-shaped noise. Target sentences were presented in front of the listener, with noise sources placed at -135° and 135°, respectively. Outcome measures were the differences in speech reception threshold using omnidirectional, fixed and adaptive beamformer microphone settings.

**Results:**

The use of directional microphones significantly improved speech reception thresholds: fixed beamformer vs. omnidirectional: 4.3 dB (95%-CI [3.1; 5.5]), p<0.0001; adaptive beamformer vs. omnidirectional: 6.1 dB (95%-CI [4.9; 7.3]), p<0.0001; and adaptive beamformer vs. fixed beamformer: 1.8 dB (95%-CI [0.7; 3.0]), p = 0.001.

**Conclusion:**

This study confirms the previously reported improvements in speech perception in noise of the fixed beamformer microphone setting and is the first to report significant improvements in speech perception in noise when applying the adaptive beamformer microphone settings of the SONNET audio processor. Cochlear implant users may be able to benefit from improved hearing performance especially in difficult listening situations.

## Introduction

Cochlear implants (CIs) present a safe and effective treatment for severe-to-profound hearing loss, and many CI users achieve remarkable levels of speech perception in quiet [1]. Nevertheless, speech perception in noise, mainly in unilaterally-aided subjects, still remains a major challenge due to factors such as the loss of fine spectral and temporal resolution and a relatively narrow dynamic range of electrical stimulation [2, 3]. Bilateral implantation results in improvement in challenging listening situations as well as improved coding strategies and the preservation of low frequency hearing [4–7]. Despite these innovations, CI users have inferior speech perception in noise compared to normal hearing subjects [8, 9]. Further refinements have been established to improve patient outcome in challenging environments, including algorithms optimizing input dynamic compression and range, automatic gain control, single channel and multi-channel noise reduction techniques. Another effective strategy to decrease the effects of competing noise on speech perception is the use of multiple microphones and beamforming algorithms. This concept is well-established for hearing aid users, with improved speech perception in noise reported for adults and young patients [10, 11]. Directional microphones that reduce the adverse effects of noise in spatially-separated target and interfering signals have previously been tested successfully in CI users [12–15]. Studies evaluating directional microphones with fixed directivity in CI patients reported improved speech reception thresholds (SRTs) between 5–7 dB compared to less directional sub-cardioid microphones [12, 13]. Fixed beamformers provide a particular directivity pattern regardless of the specific sound field characteristics, and can be used for instance to mimic the directivity of the human ear. The use of adaptive beamforming technology may result in even better speech perception [13] since the algorithm constantly detects the direction the noise is coming from and adapts the polar pattern to attenuate the sound coming from this direction.

The aim of this study was to evaluate both the fixed beamformer and the adaptive beamformer settings of the new MED-EL SONNET audio processor in regard to effects on speech perception in noisy environments with spatially separated sound sources.

## Materials and methods

### Participants

18 postlingually deafened adult CI recipients implanted with MED-EL devices participated in the study. To ensure a representative sample of the adult CI users treated at our clinic, subjects with different ear conditions and etiologies were recruited. This included 12 bimodal, two bilateral, two unilateral (bilaterally deaf) and two patients with single-sided deafness. Age ranged between 18 and 76 years (mean: 54.6), and mean implant experience was 60.3 months. Demographic details and etiology are presented in Table 1. The study protocol was approved by the Ethics Committee of the Medical University of Vienna and all subjects gave their written informed consent prior to participation.

**Table 1:** Demographic characteristics of the study population. Subject demographic data.

ID	Gender	Age (years)	Ear status (left–right)	Tested side	Implant experience (months)	WRS @ 65 dB SPL (% percent)	Etiology
1	F	45	CI-NH	L	43	75	progressive
2	F	45	CI-HA	L	7	45	progressive
3	M	62	CI-NH	R	12	40	Meniere’s disease
4	F	49	HA-CI	R	8	80	progressive
5	F	18	CI-CI	R	164	65	progressive
6	M	54	CI-HA	L	34	40	sudden hearing loss
7	F	50	CI-HA	L	41	50	tympanosclerosis
8	F	76	HA-CI	R	105	40	progressive
9	M	58	HA-CI	R	139	60	progressive
10	F	52	*Deaf*-CI	R	61	70	progressive
11	M	52	HA-CI	R	73	60	otosclerosis
12	F	24	HA-CI	R	37	55	progressive
13	F	61	CI-CI	L	114	55	progressive
14	M	76	CI-HA	L	83	55	progressive
15	M	67	HA-CI	R	6	60	progressive
16	F	54	*Deaf*-CI	R	105	55	progressive
17	F	76	HA-CI	R	46	35	progressive
18	M	64	HA-CI	R	8	45	progressive

F = female; M = male; CI = cochlear implant; HA = hearing aid; NH = normal hearing; WRS = word recognition score with CI; *Deaf* = profoundly deaf; L = left; R = right.

### Fitting

All subjects were tested with their individual fitting map. All the devices investigated were SONNET audio processors, therefore subjects either used their own SONNET audio processor or a SONNET audio processor provided by our clinic for the duration of testing if they were using an audio processor without microphone directionality. Microphone protectors were checked and replaced, if necessary, prior to testing. Other features of the audio processor like front-end *wind noise reduction (mild)* and *automatic gain control (*compression ratio: 3:1*)* were set to the same default settings for all subjects. Adaptive microphone directionality was set to “always directional”. Three different directional microphone settings were created: (1) “omnidirectional”microphone mode (OMNI), (2) “natural” mode that mimics the directivity of the human pinna based on fixed beamforming technique (FBF), and (3) “adaptive” mode applying an adaptive beamforming scheme (ABF). The FBF generates a static directivity pattern where sounds from the rear hemisphere are attenuated. This is achieved by adding a specific delay between the microphone channels and subsequent summation of the signals. The degree of attenuation is frequency-dependent. For lower frequencies, the directivity pattern is similar to OMNI; with increasing frequency the attenuation increases, with the highest attenuation reached at an angle of 135°. The ABF uses an adaptation algorithm to estimate the position of the noise source, which is based on the particular sound field characteristics. The direction of attenuation is then adjusted accordingly.

### Speech tests

**Fig 1:**
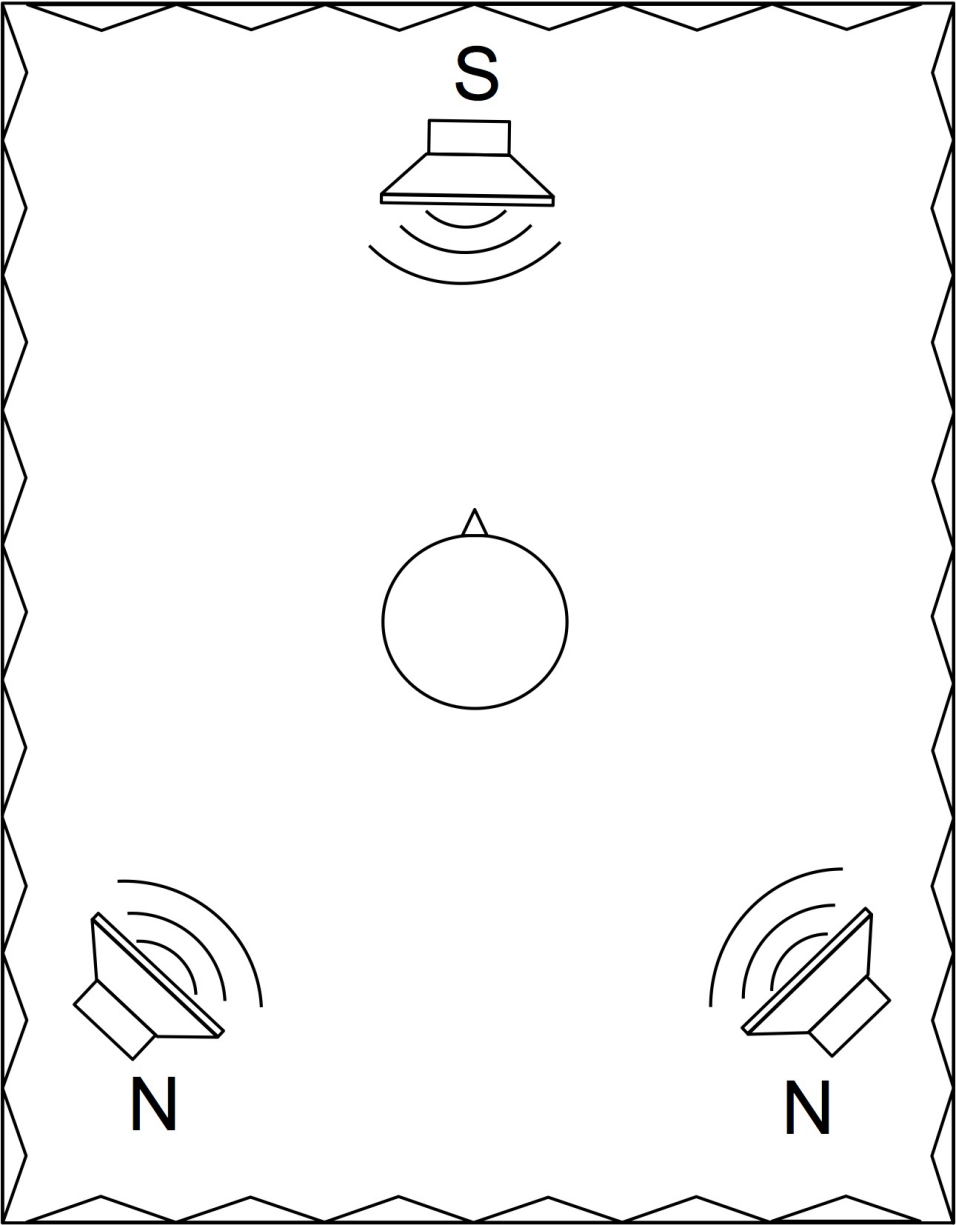
Experimental setup. All loudspeakers were placed in the horizontal plane approximately at ear height. Speech (S) was presented from 0° azimuth, noise sources (N) were placed at ±135° azimuth. The distance between sound sources and the center of the listener’s head was 130 cm.

**Fig 2:**
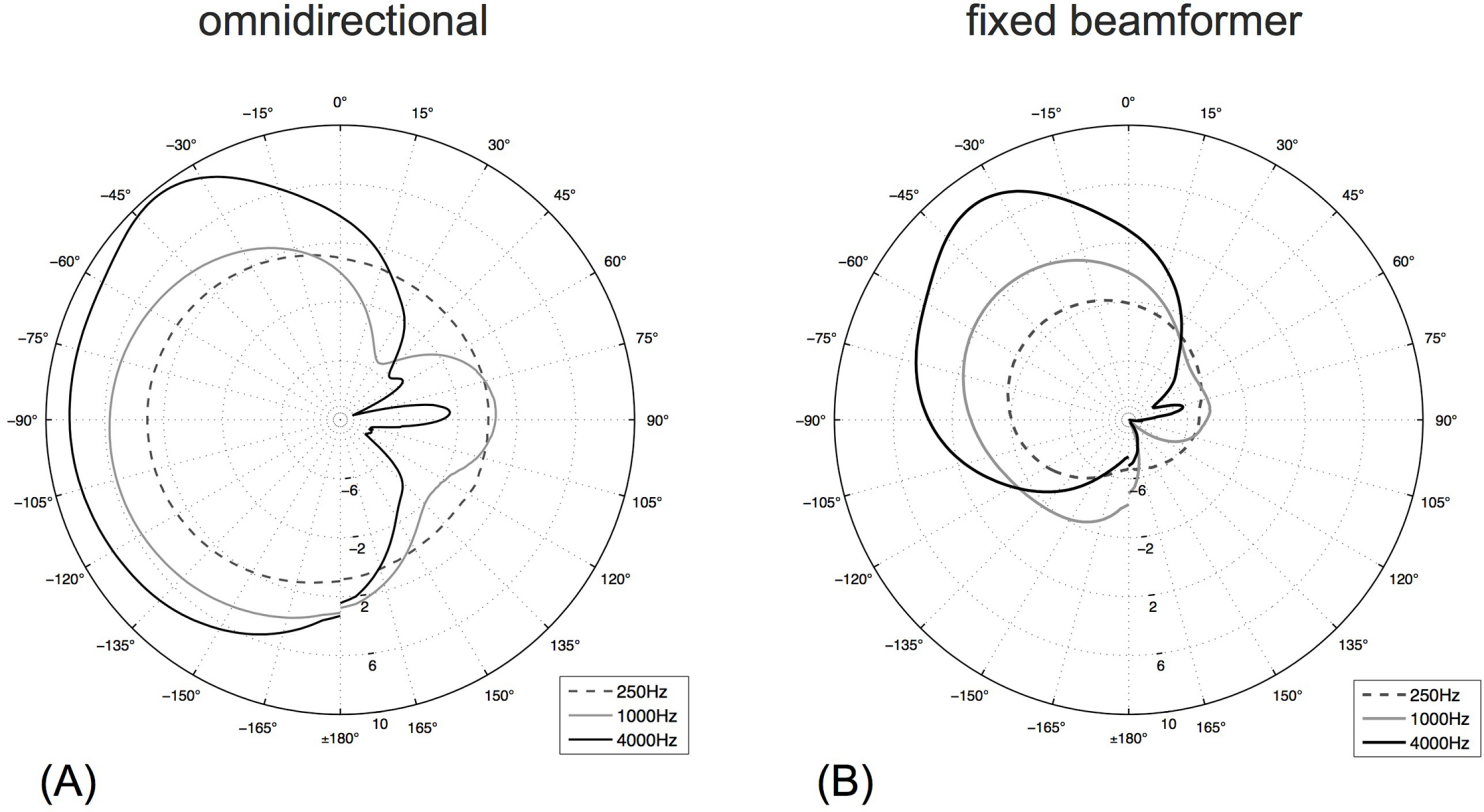
Microphone directivity patterns. Microphone directivity patterns of the SONNET audio processor at different frequencies for (A) the omnidirectional setting, and (B) the fixed beamformer setting, normalized to 0 dB for 0° azimuth at 1000 Hz. Measurements were performed in an anechoic chamber with the audio processor placed on the left ear of a KEMAR mannequin.

Speech perception in noise was evaluated with the Oldenburg Sentence Test (OLSA), an adaptive, closed-set, German language sentence test [9, 16, 17]. The measured SRT quantifies speech perception in noise, with results in dB signal-to-noise ratio (SNR) at which 50% of the presented words are perceived correctly. The target sentences, presented by a male speaker, consist of five randomly selected words from a list of 10 possible choices. The noise signal (OLSA noise) consists of 30 randomly time-shifted OLSA sentences and therefore exhibits spectral characteristics similar to the short-time frequency spectrum of the target sentences [9]. The speech tests were performed in a sound-isolated chamber (3.15 × 3.10 ×2.10 m) with a reverberation time T_60_ of 0.15 s at 500 Hz. Bilaterally implanted subjects were tested in their better ear and were instructed to deactivate the contralateral device; in patients with single-sided-deafness (SSD) or bimodal hearing, the contralateral ear was plugged and covered with earmuffs to minimize the effect of residual hearing. Measurements and signal playback were controlled with MATLAB (The MathWorks, Inc.); speech signals were presented over a WESTRA LAB 501 loudspeaker and JBL Control 1 pro loudspeakers were used for noise playback. Speech was presented from 0° azimuth at a distance of 130 cm from the subject’s head, with noise sources placed at ±135° azimuth (Fig 1) at the same distance; all sound sources were located in the horizontal plane at ear height. Noise sources were calibrated to ensure the same output sound pressure level (SPL). In a second step, the overall noise field level was adjusted to reach a certain SPL at the listening position. The noise level was constant at 65 dB, and the speech level was adapted depending on the subject’s performance based on the adaptation scheme of the OLSA. The initial SNR was +10 dB in all subjects. Subjects were advised to avoid head movements during signal playback. The choice of the noise source locations was based on the measured directivity patterns of the SONNET audio processor, illustrated in Fig 2. It was assumed that the difference in sensitivity between OMNI and FBF would correlate with the difference in SRT between the settings. Therefore, the noise sources were placed to expect a potentially large impact of the FBF setting. Two noise sources were chosen instead of a single noise source situation as this setup represents a more realistic scenario as well as an additional challenging situation for the ABF scheme. The average level difference due to the head shadow between ipsilateral and contralateral noise source was 4.2 dB (n = 4) at the position of the SONNET microphones.

The sensitivity pattern of the ABF structure depends on the instantaneous sound field characteristics and thus cannot be generalized by means of sensitivity measurements. However, if the spatial separation between speech source and noise sources is sufficient, a positive effect on the SRT can be expected. To ensure accurate assessment of the ABF impact, the experimental setting had to fulfill certain requirements: 1) the noise had to be continuously presented to provide sufficient adaptation time for the ABF algorithm. And 2) the noise signals had to be uncorrelated to ensure that they were treated as independent sources by the ABF algorithm. This was achieved by introducing a random phase shift between the two noise channels.

### Study design

The experiment was conducted in a single-blinded manner in an acute setting. Prior to testing, two training lists consisting of 20 sentences were presented to the participants to allow them to familiarize themselves with the test procedure and the stimuli material. This was followed by the presentation of two more lists (20 sentences) for each testing condition, to assess SRTs. To minimize both learning and fatigue effects, sentence lists and testing order with respect to the microphone setting were randomized.

### Statistical analysis

The SRT values from both test lists for each condition were included in an ANOVA model in regard to the fixed factor “microphone setting” and the random block factor “patient”. The Tukey-Kramer method was used to perform multiplicity-adjusted, pairwise comparisons between group means. Group differences are described by the mean difference with adjusted 95% confidence intervals (CI).

All p-values are results of two-tailed tests; a p-value of <0.05 was considered statistically significant. Statistical analysis was performed with SAS (SAS Institute Inc. (2002–2017), Cary, NC, USA).

## Results

**Fig 3:**
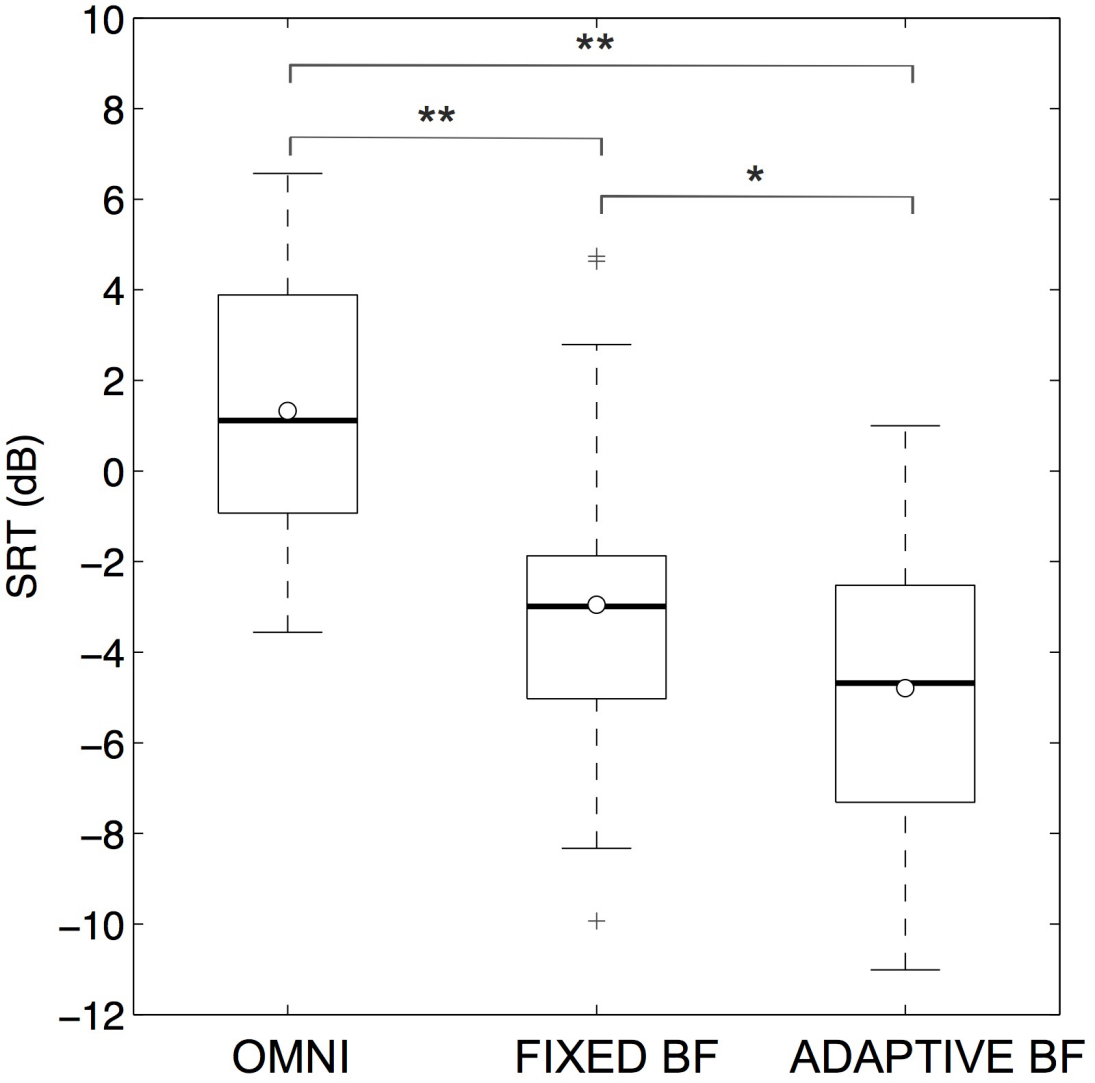
SRTs. Measured SRT (dB) for omnidirectional setting, fixed beamformer setting and adaptive beamformer setting. * indicates p = 0.001; ** indicates p<0.0001.

**Fig 4:**
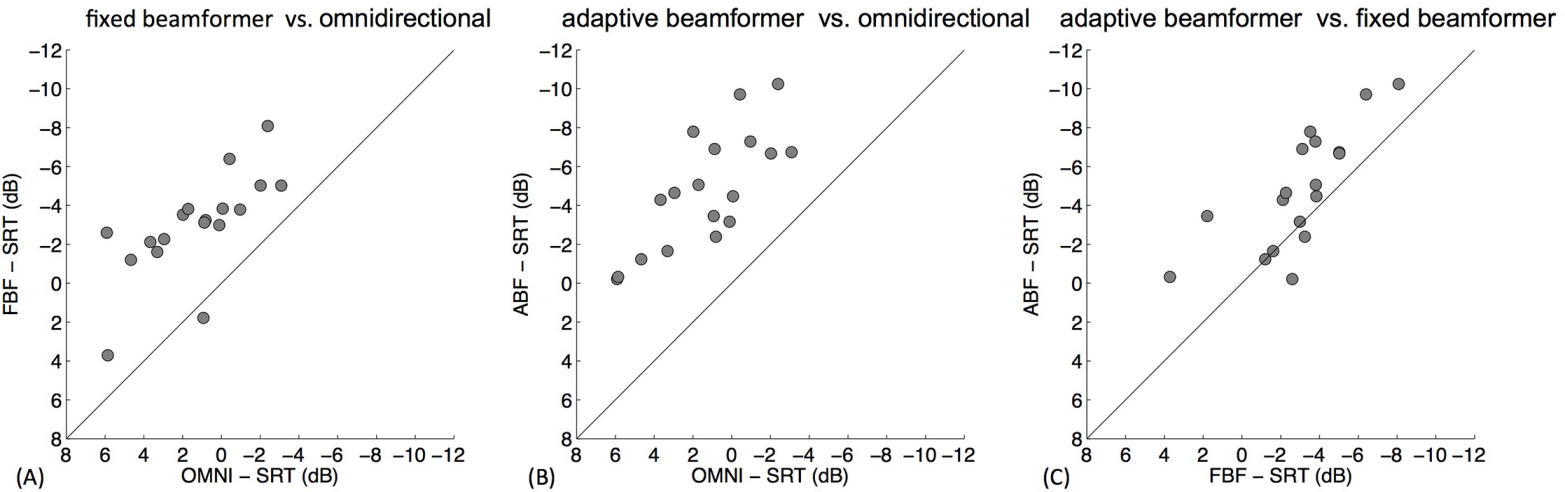
Individual differences in terms of SRT for each subject. (A) Fixed beamformer (FBF) vs. Omnidirectional microphone (OMNI), (B) Adaptive Beamformer (ABF) vs. OMNI, and (C) ABF vs. FBF.

The SRT results for all conditions are shown in Fig 3. Analysis of variance revealed a highly significant effect (p<0.0001) of the microphone setting. Both directional microphone conditions showed statistically significant improvements in SRTs compared to the OMNI condition. With the FBF setting the SRT improved on average by: 4.3 dB (95%-CI [3.0; 5.5]), p<0.0001; for the ABF condition the improvement was 6.1 dB (95%-CI [4.9; 7.3]), p<0.0001. SRTs of ABF compared to FBF improved on average by 1.8 dB (95%-CI [0.7; 3.0]), which was also statistically significant (p = 0.001). No statistically significant learning effects between the first and second run were observed (p = 0.59). Fig 4 illustrates the data differences for the SRT (average of both runs) on an individual level. The FBF format improved speech perception in noise in all but one of the participants (Fig 4A). In the ABF setting, all patients showed improved SRTs as compared to the omnidirectional microphone setting (Fig 4B). In comparison to the FBF scheme, the ABF technique improved SRTs in 14 patients (77.8%), showed comparable results in 2 cases (11.1%) and caused deterioration in SRTs in 2 CI users (11.1%) (Fig 4C).

## Discussion

The aim of this study was to evaluate the new MED-EL SONNET cochlear implant audio processor’s fixed and adaptive beamformer techniques for potential benefits to speech perception in noise. The statistically significant improvements of 4.3 dB in SRTs for the FBF scheme and 6.1 dB for the ABF technique are in the range of the benefits reported in the literature [12–15, 18, 19]. However, it should be noted that these results are difficult to compare due to inconsistencies in technical setups and applied hearing tests. Further differences between the studies include the evaluated speech processors and coding strategies, spatial conditions, spectral and temporal noise characteristics, speech testing paradigms, and subjects’ ear status. Even though no discrimination function was measured for the particular spatial setup used in our study, the benefit in speech perception provided by beamforming can be considered relevant. The slope of the discrimination function for CI users (13.6%/dB) in the co-located masker condition S0N0 was smaller than the slope of the S0N0 condition for normal hearing subjects (17.1%) [9, 20]. In other studies testing normal hearing subjects in modified setups, Rader et al. (2008) measured a slope of 14%/dB in a multi-source noise field and Wagener (2004) 11.3%/dB in quiet [21, 22]. When attempting to link the reported SRTs and the published slopes of the discrimination function, it is important to keep in mind that the discrimination function of the OLSA exhibits a high variability between CI users. It has been demonstrated that in this specific patient population the discrimination function is steeper in better performers, who also exhibit a higher test-retest reproducibility [20]. It should also be highlighted that the setting used in this study may overestimate the benefit in real life, as more reverberant environments, different noise signal characteristics and moving noise sources potentially diminish the beneficial effect of beamforming algorithms [15, 23]. The spatial setup used in this study represents a rather straightforward situation for beamformers, since the noise sources were placed at locations where high attenuation may be expected. Furthermore, microphone aging and environmental influences like dust could decrease the beamformer performance in everyday life. CI users benefited from FBF and ABF characteristics immediately without the necessity of adaption time. In the given setting both FBF and ABF provided a very consistent and predictable benefit. In the ABF scheme, all patients, regardless of etiology or CI experience, showed improved SRTs as compared to the omnidirectional microphone settings. Razza et al. evaluated the impact of FBF and ABF settings of the Nucleus 5 CP810 sound processor in a S_0°_N_45°_ scenario and, in contrast to our results, did not find a significant difference [24]. Apart from the fact that the directional sensitivities of the CP810 beamformers are slightly different compared to the SONNET, this is most likely due to the different spatial setups of speech and noise sources. This illustrates a potential weakness of adaptive beamforming algorithms: depending on the listening environment, they can outperform both omnidirectional microphones and directional microphones with fixed directivity, but they could theoretically also deteriorate the target signal in situations where noise and target signal occur at similar angles. Additionally, they might deteriorate sound localization and decrease awareness for environmental sounds such as cars. In fact, the ABF characteristics implemented in the SONNET are only intended to suppress noise sources from the rear hemisphere. An additional feature of the SONNET processor which is intended to improve speech perception in noise is the automatic switching between OMNI and ABF settings, depending on the input signal level. When activated, the ABF is applied for high-input levels only, i.e., when the listening environment is potentially difficult for the CI user. Because adaptive microphone directionality was set to “always directional”, this feature was not tested in this study.

It is worth noting that the ABF performs significantly better than the FBF in our particular test scenario with two noise sources, although a two-channel ABF is designed to attenuate only a single direction. This might be due to the attenuation of the second noise source because of the head shadow effect, which results in the selection of the ipsilateral noise source as the loudest direction.

This study evaluated the benefits of beamforming with a single CI. Since complex binaural beamforming algorithms are already established for hearing aids and such algorithms have also been successfully tested in CI users [25], the use of beamforming in bilaterally implanted patients as well as the use of even more sophisticated bilateral beamforming algorithms might further improve speech perception in noise in the near future.

## Conclusion

CI audio processors equipped with directional microphones improve speech reception in noise substantially, given that speech and noise sources are spatially separated. In the presented scenario, the ABF algorithm of the MED-EL SONNET audio processor yielded significantly better results compared to the FBF algorithm and the omnidirectional microphone. The FBF algorithm produced significantly better results than the omnidirectional microphone setting. However, emphasis needs to be drawn to the fact that directional microphones may also decrease the awareness of sudden sound sources like approaching cars. Furthermore, adaptive beamforming may also deteriorate speech perception in certain listening situations. Future studies are needed to evaluate the benefit of the ABF algorithm in everyday life. Until then, the FBF scheme could be the recommended standard setting for hearing in noise in CI patients, to allow them to benefit from this promising technology.
